# Use of beneficial bacterial endophytes: A practical strategy to achieve sustainable agriculture

**DOI:** 10.3934/microbiol.2022040

**Published:** 2022-12-27

**Authors:** Rudoviko Galileya Medison, Litao Tan, Milca Banda Medison, Kenani Edward Chiwina

**Affiliations:** 1 College of Agriculture, Yangtze University, Jingzhou Hubei 434025, China; 2 Department of Horticulture, University of Arkansas, Fayetteville, AR 72701, USA

**Keywords:** beneficial endophytic bacteria, biological control, environmental stresses, plant growth promotion, synthetic chemicals

## Abstract

Beneficial endophytic bacteria influence their host plant to grow and resist pathogens. Despite the advantages of endophytic bacteria to their host, their application in agriculture has been low. Furthermore, many plant growers improperly use synthetic chemicals due to having no or little knowledge of the role of endophytic bacteria in plant growth, the prevention and control of pathogens and poor access to endobacterial bioproducts. These synthetic chemicals have caused soil infertility, environmental contamination, disruption to ecological cycles and the emergence of resistant pests and pathogens. There is more that needs to be done to explore alternative ways of achieving sustainable plant production while maintaining environmental health. In recent years, the use of beneficial endophytic bacteria has been noted to be a promising tool in promoting plant growth and the biocontrol of pathogens. Therefore, this review discusses the roles of endophytic bacteria in plant growth and the biocontrol of plant pathogens. Several mechanisms that endophytic bacteria use to alleviate plant biotic and abiotic stresses by helping their host plants acquire nutrients, enhance plant growth and development and suppress pathogens are explained. The review also indicates that there is a gap between research and general field applications of endophytic bacteria and suggests a need for collaborative efforts between growers at all levels. Furthermore, the presence of scientific and regulatory frameworks that promote advanced biotechnological tools and bioinoculants represents major opportunities in the applications of endophytic bacteria. The review provides a basis for future research in areas related to understanding the interactions between plants and beneficial endophytic microorganisms, especially bacteria.

## 1. Introduction

Recently, there have been studies that focus on the use of microorganisms to enhance nutrition element availability in the soil and control plant disease without the use of synthetic fertilizers, pesticides or herbicides. One of the typical microorganisms that have the potential to promote plant growth and protection from pathogens is a group of beneficial bacterial communities known as endophytes that are harbored in plant organs such as roots, leaves, stems shoots and flowers [1]–[3]. Research has shown that some bacterial endophytes also colonize agronomic crops and play a role in providing and enhancing nutrient availability and biological control mechanisms against pathogens and insect pests [4],[5]. Endophytic bacteria are not limited to a single function, but have multiple plant growth-promoting and biocontrol traits that can be released simultaneously [6]. For example, endophytic *Paenibacillus polymyxa* was able to fix nitrogen, solubilize phosphorous, synthesize phytohormones and display biocontrol properties against pathogenic fungi [7].

Endophytic bacteria use various physical, molecular and biochemical mechanisms to perform and display various growth and biocontrol traits [8],[9]. The ability to exhibit most of the plant growth-promoting and biological control traits qualifies the specific endophytic bacteria to be a reliable agent in plant growth, reproduction and protection; therefore, such bacteria can be researched further and formulated for commercial purposes [10],[11]. Most of the beneficial endophytic bacteria absorb very important organic acid-metal complexes such as copper, iron, zinc and magnesium. The penetration of endophytic bacteria into the plant roots allows plants to extract these metals from the microbes [12]. On the other hand, inoculating plants with endophytic bacteria could inhibit disease symptoms initiated by disease-causing organisms such as insects, nematodes, fungi bacteria and viruses [13],[14].

There have been few applications and formulations of endophytic bacteria in agriculture. Furthermore, many growers continue with the heavy use of synthetic chemicals because of a poor understanding of the roles of endophytic bacteria in plant growth promotion and plant health improvement. There is also a perspective that most of the microbes are pathogenic to plants. These challenges show that there is still a gap between the research and the normal use of endophytic bacteria and their products by farmers. Therefore, this paper is aimed to review the progress in the research on the major roles played by endophytic bacteria in alleviating biotic and abiotic plant stresses, increasing plant growth and yield performance and biologically controlling major plant pathogens. The review will provide opportunities, gaps in the bacterial endophytes research and the way forward to fully utilize these untapped microorganisms. Based on our knowledge, this review will provide a basis for future research areas dedicated to understanding the interactions between plants and their endo-microbes.

## 2. Promotion of plant growth and development

### 2.1. Nitrogen fixation

Recently, there have been many studies about nitrogen-fixing bacteria focusing on applying the same concept of symbiotic associations that occur in legumes to non-leguminous plants such as maize, sorghum wheat and sugarcane [15]. Moreover, even those plants under various environmental stress can benefit from the biologically fixed nitrogen by endobacteria. The efficiency of nitrogen fixation by other endophytes cannot surpass that fixed by the *Rhizobium* sp. bacteria in leguminous plants [16]. However, this nitrogen is of paramount importance, especially in host plants that grow in limited nitrogen soils, as has already been proved by many researchers. Recently, nitrogen-fixing diazotroph bacteria (*Gluconacetobacter diazotrophicus*) have been isolated from the tissues of the sugarcane plants. This bacterium was able to grow and fix nitrogen, thus causing postulation that these bacteria can satisfy the nitrogen requirements of its host plant [17],[18].

### 2.2. Solubilization of phosphate

Phosphate is the precursor for the synthesis of various enzymes responsible for various plant physiological processes, in addition to aiding plant disease resistance [19]. To be changed into an accessible soluble form, organic and inorganic phosphates need to undergo processes of solubilization and mineralization with the aid of bacterial enzymes known as phosphatases that are controlled by the presence of genes [20]. During phosphate solubilization by phosphate-solubilizing bacteria, chelators that are organic acids are produced and help to displace metals [21]. Research has indicated that more than 20 copies of genes responsible for phosphate solubilization were found in the non-phototrophic endobacteria metagenome. Endophytic bacteria that solubilize phosphate into an accessible form help their host organisms to grow and survive even in poor environmental conditions and improve growth and yield performance even when inoculated in crop plants [2],[22]. By contributing the major nutrition elements to plants, phosphate-solubilizing bacteria contribute more to the functions, diversity and ecology of plants in the ecosystems. However, there is a need to explore more of the endophytic phosphate-solubilizing bacteria, from the molecular level to their practical applications, as they are very crucial in sustainable agriculture and environmental protection, and very little research has been done to date.

### 2.3. Solubilization of potassium

Most of the potassium-solubilizing bacteria lives in the soil [23]. However, some endophytic bacteria are reported to have the ability to solubilize the unavailable potassium into accessible forms. As a result, endophytic bacteria have attracted attention in agriculture for soil root inoculation because of their capacity to penetrate and colonize root interiors [24]. Potassium-solubilizing endophytic bacteria work by synthesizing and discharging organic acids such as oxalic acid, tartaric acid, malic acid and gluconic acid. These acids break the insoluble minerals from various minerals mentioned previously to release accessible soluble potassium [25],[26]. Potassium-solubilizing endophytic bacteria have also been reported to alleviate other environmental stresses, such as salt stresses, and improve production in general [27],[28]. Unfortunately, most of the endophytes that have been isolated and evaluated were targeted for the evaluation of other growth promotion traits such as nitrogen and indole-3-acetic acid, leaving out the role that potassium plays in plant growth and protection.

### 2.4. Uptake of iron nutrition element

Under iron-limiting conditions, some bacteria release low molecular weight iron-chelating molecules called siderophores that exist in various varieties [29]. Siderophores are described as useful peptide chains and functional groups that allow iron ions to bind [30]. Siderophores have been demonstrated to be the source of iron for plant nutrition. Siderophore-producing bacteria have mechanisms that facilitate the availability of iron in very iron-limiting environments. These bacteria strains have outer membrane proteins on their cell surface that transport iron complexes, making the iron available for metabolic processes. Siderophores have a high affinity for iron and bind Fe^3+^, which is later assimilated by root hairs [31]. Many researchers report both the nutrition and biocontrol significance of siderophores; therefore, the provision of this important endophytic bacteria trait in plant growth and protection cannot be undermined. Siderophore produced by *Streptomyces* spp., an endophyte from the roots of a Thai jasmine rice plant, remarkably promoted plant growth and improved root and shoot length and overall yield [32].

### 2.5. Zinc solubilization

Zinc is one of the important trace elements needed by plants and other living things. It influences metabolism and enzymatic activities in plants, although it is a trace element. As a result, the absence of zinc elements in plants is easily noticed from the perspective of the field to the products of crops that lacked zinc elements. Some of the bacterial zinc solubilizers include *Gluconacetobacter*, *Bacillus*, *Acinetobacter* and *Pseudomonas* [33]. Zinc-solubilizing bacteria provide a sustainable and healthy alternative for supplying and converting applied inorganic zinc into a form that can be accessed by plant roots [34]. The inoculation of zinc-solubilizing bacteria has been reported to promote plant growth and yield performance, as well as to improve the nutrition value of maize and rice as part of bio-inoculants for biofortification [35],[36]. Zinc-solubilizing endophytic bacteria *Pseudomonas* sp. MN12 were used in combination with other zinc-supplying materials and proved to improve the grain biofortification of wheat [37]. Endophytic bacteria isolated from soybean and summer mungbean were able to solubilize zinc, and researchers have found that *Klebsiella* spp. and *Pseudomonas* spp. produced other plant growth-promoting components such as phosphate and indole-3-acetic acid [38]. With these few given examples, zinc-solubilizing endophytic bacteria require more attention in research and practical applications to improve the plant growth of the most important crops and enhance their nutritive value such that the end will ensure food and nutrition security, as well as environmental protection.

### 2.6. Synthesis of phytohormones

The use of plant growth regulators from beneficial microorganisms is one promising strategy to enhance plant growth under normal or stressful conditions [39]. The most notable plant growth-promoting hormones that can be synthesized by bacteria include indole-3-acetic acid, zeatin, abscisic acid, cytokinins and gibberellic acids and ethylene [40]. Indole-3-acetic acid is one of the mechanisms which bacteria use to interact with plants, signaling molecules in bacteria and influencing plant growth and development [41]. Indole-3-acetic acid produced by endophytic bacteria has also been reported as a plant defense mechanism against pathogens that would otherwise cause diseases in plants [42]. Gibberellic acid produced by *Azospirillum* spp., an endophyte, was found to contribute to alleviating drought stress and enhancing plant growth in maize (*Zea mays*. L) [43]. While gibberellic acids are known to improve plant growth and development, some researchers have reported that the hormone has some root growth-inhibiting influence through the gibberellic DELLA-repressing signaling system [44]. However, sufficient synthesis and production of gibberellic acids in bacteria has major advantages, in terms of plant growth and development, over the growth inhibitory influence that this hormone can display [45].

**Table 1.** Examples of some of the endophytic bacteria that have so far been isolated, identified and evaluated for their plant growth promotion and biocontrol effects.

Role	Bacteria	references
Nitrogen fixation	*Pseudomonas* spp., *Herbiconiux solani* SS3, *Flavobacterium aquidurense* SN2r, *Rhizobium herbae SR2r*., *Paenibacillus polymyxa* P2b-2R, *Pseudomonas protegens* CHA0-retS-nif	[46]–[49]
Phosphorous solubilization	*Pseudomonas* spp. *Burkholderia* spp, *Paraburkhoderia, Novosphingobium, Ochrobactrum, Paenibacillus polymyxa, Bacillus* sp., *Rahnella Pantoea vagans* MZ519966, *Pantoea agglomerans* MZ519970, *Pseudomonas aeruginosa* KUPSB12	[50]–[54]
Potassium solubilization	*Paenibacillus polymyxa, Bacillus* sp.*, Burkholderia* sp. FDN2-1, *Alcaligenes* spp., *Enterobacter* spp.	[24],[51],[55]
Zinc solubilization	*Bacillus* spp., *Arthrobacter* sp., *Klebsiella* spp., *Pseudomonas* spp.	[38],[56]–[58]
Hormones (indole-3-acetic acid jasmonic acid, salicylic acid, gibberellins, ethylene)	*Klebsiella* sp., *Enterobacter* sp., *Bacillus amyloliquefaciens* RWL-1; *Bacillus* sp. PVL1, *Bacillus* sp. DLMB, *Bacillus* sp. MBL_B17, *Bacillus subtilis* MBL_B13, *Leifsonia xyli* SE134, *Bacillus subtilis* LK14,	[59]–[66]
Siderophores and competition for nutrition and space	*Bradyrhizobium* sp.(vigna), *Pseudomonas tolaasii* ACC23, *Mycobacterium* ACC14 *Pseudomonas fluorescens* G10, *Mycobacterium* sp. G16, *Methylobacterium* spp., *Xanthomonas* spp.	[16],[67]–[70]
Induced Systemic Resistance	*Parabukholderia* sp. *Pseudomonas* sp, Burkhoderia phytofirman PsJN	[71]–[74]
Lytic Enzymes {chitinases, proteases, cellulases, hemicellulases, 1, 3-glucanases; pectinases,	*Serratia proteamaculans* 33x, *Bacillus pumilis* JK-SX001, *Paenibacillus polymyxa* GS20, *Bacillus* sp. GS07	[75]–[77]
Antibiotics (Bacillomycin 2,4-diacetylphloroglucinol, fencing, cyclic lipopeptides (surfactin, iturin), and pyocyanin}	*Bacillus subtilis* fmbj, *Bacillus subtilis* CPA-8, *Bacillus subtilis* AU195	[73],[78]–[80]
Volatile Organic Compounds (2,3-butanediol, acetoin, 2-Hexanone, sulfur-containing compounds, 2-Heptonone, 3-methybutan-1-ol, Dodacanal, 3-methylbutanoic acid, and 2-methylbutanoic acid, 3-Methylbutan-1-ol)	*Bacillus amylolicefaciens* ALB629 and UFLA285, *Enterobacter* TR1, *Bacillus* spp. *Bacillus Velenzensis* 5YN8, *Bacillus Velenzensis* DSN012	[81]–[83]

## 3. Biocontrol of plant pathogens

Endophytic bacteria are reported as suitable biocontrol agents owing to their ability to be sustainably transferred to the next generation [13],[84],[85]. The other advantage of endophytic bacteria in biocontrol is that they do not compete with plants for space and nutrition, but contribute to and improve the health of their host plants [86],[87]. Some of the endophytic bacteria with biocontrol properties have well been documented in a previous review [88]. Endophytic bacteria of genera *Arthrobacter, Pseudomonas*, *Serratia*, *Bacillus* and *Curtobacterium* [89],[90] are the best representatives that are used in the biocontrol of plant pathogens and diseases. Usually, after their isolation from the host plant, endophytes are tested by performing dual plate assays and a genetic screening approach [90],[91]. *Bacillus* spp. have been reported to be good biocontrol agents because of their ability to synthesize a wide range of biologically active molecules that are potent inhibitors of plant pathogens. Some seed associated endophytic bacteria, i.e., *Bacillus subtilis*, *Bacillus velezensis*, *Leuconostoc mesenteroides*, *Lactococcus lactis* and *Bacillus amyloliquefaciens*, were all used to treat bacterial wilt of tomato, and all isolates were able to exhibit biocontrol properties [92]. Other associated bacterial endophytes have been noted to produce secondary metabolites [93] that might play a role in the biocontrol of plant pathogens. Moreover, *Bacillus velezensis* 8-4 was found to inhibit potato fungal pathogens such as *S. galilaeus*, *Phoma foveat*, *Rhizoctonia solani*, *Fusarium avenaceum and Colletotrichum coccodes* in both in vitro and field experiments [94]. These are just a few examples; however, endophytic bacteria have been used in many applications to control the introduction and growth of notable plant pathogens [95].

Endophytic bacteria have several mechanisms to inhibit and control the growth of plant pathogens, which some researchers have documented [96],[97]. Most notable is the presence of genes responsible for particular biocontrol traits such as antibacterial and antifungal metabolites that have been identified in the whole genomes of some endophytic bacteria [98]–[100] Some endophytic bacteria help their host to develop induced systemic resistance (ISR) that comes when plants successfully activate their defense mechanism in response to primary infection by a pathogen [84]. The production of siderophores and antimicrobial compounds as a form of mechanism for biocontrol has so far been well documented in various research manuscripts [101]. Therefore, endophytic bacteria isolates can be commercially formulated into biopesticides to help protect plants while ensuring a healthy environment [102] Some of the mechanisms have briefly been described as researched in the past few years.

### 3.1. Upregulation of host defense genes

During the primary infection by pathogens, most plants develop and activate various defense mechanisms. Furthermore, plants interact with endophytic bacteria and activate plant resistance against pathogens such as bacteria, fungi and viruses. This type of resistance is known as ISR. The ability of beneficial microbes such as endophytic bacteria to initiate ISR is host-specific and requires full colonization of a type of bacteria to their host plant [103]. The endophytic traits such as the production of volatile compounds, bacterial flagellation and the production of lipopolysaccharides and highly sensitive hormones all determine the development of ISR in plants [104].

The pathogenesis-related genes and the jasmonic/ethylene-dependent genes induce systemic resistance which is triggered by endophytic and other plant growth-promoting bacteria [73],[105],[106]. Under normal circumstances, the endophytic bacteria in plants trigger a very minimal level of systemic acquired resistance as compared with the moment that a pathogen has been introduced. Once the pathogen has been encountered, plants with endophytes exhibit a high level of systemic acquired resistance and jasmonate and ethylene genes are overexpressed, hence triggering biocontrol mechanisms. Endophytic bacteria have an advantage in that they induce both the systemic acquired resistance and jasmonic/ethylene-dependent ISR that helps plants to simultaneously resist bacterial and fungal pathogens such as *Pectobacterium carotovorum* and *Fusarium oxysporum* [107].

Endophytic bacteria alleviate the adverse and detrimental effects of plant pathogens by actively inducing the resistance mechanisms in plants. It includes the activation of idle and latent defense mechanisms when the pathogenic stimuli are sensed; usually, this process is controlled by the complex networks of signaling pathways [72], [108]. For example, *B subtilis* GBO3 and *B. amyloquefaciens* IN937a produced volatile compounds that trigger the ISR against *Erwinia carotovora*; the research gave proof that the signaling pathway that was activated by the volatile compound from *B. subtilis* GBO3 is dependent on the ethylene and independent from salicylic and/or jasmonic acid signaling pathways [109], thus giving the difference between systematic acquired resistance and ISR [110]. As part of the mechanism to trigger ISR defense, endophytic bacteria may cause the cell wall of plant cells to strengthen upon the introduction of a pathogen, thus providing a barrier for pathogens. Endophytic bacteria may also modify the physiology and alter metabolic processes in plants that will result in the improved synthesis of plant defense secretions [111]–[113].

### 3.2. Competition for nutrition and space

While siderophores have been characterized to provide iron nutrition to plants, there is enough evidence that siderophores help to control the plant root pathogens by outcompeting them on limited available iron nutrition elements [104]. As described before, bacterial endophytes produce siderophores that have a strong appetite for iron elements in the rhizosphere. Competition for iron ions is one way in which biocontrol endophytic bacteria use against pathogenic fungi [114]. Siderophores bind the Fe^+3^, rendering it unavailable to the fungal pathogens that produce siderophores with less affinity for iron nutrition [104],[115]. During limited iron nutrition, root endophytic bacteria may produce siderophores that enable plant roots to make full use of the little available iron nutrition element. This makes the harmful microbes such as pathogenic fungi starve and inhibits them from causing harm to plant hosts. In summary, the production of siderophores prevents the introduction of pathogens to plants and limits their growth by outcompeting them for iron and other nutrition elements in a given ecological substrate [116].

### 3.3. Production of antibiotics

In plants, antibiotics function as antifungal, antiviral, phytotoxic antioxidant, antitoxic and antihelminthic compounds against specific pathogens. Endophytic bacteria are known to be good sources of antibiotics [117]. Usually, there must be at least one antibiotic biosynthesis-related gene that would facilitate the ability of a particular endophytic bacteria to synthesize antibiotics [118],[119]. For example, streptomyces NRR 3052, an endophyte isolated from the medicinal plant *Kennedia nigriscans* produced high-activity munumbicin antibiotics that act as plant pathogenic bacteria and fungi [120]. The ability to produce very active antibiotics by the endophytic bacteria provides a cheap source of biocontrol agents for sustainable agricultural production and environmental management.

### 3.4. Volatile organic compounds

Volatile organic compounds are signaling substances that intermediate the interaction between a plant and microbes. Volatile organic compounds are very important, as they help in the inhibition of plant-pathogen growth and induce systematic resistance in a host plant [116],[121]. Like other bacteria, endophytic bacteria may produce volatile organic compounds such as 2,3-butanediol, acetoin, 2-hexanone, sulfur-containing compounds, 2-heptonone, 3-methybutan-1-ol and dodacanal. These volatile organic compounds are formed during the metabolism of bacteria, and in the presence of stimuli that influence the internal and external conditions of the bacteria [122]. The availability of specific genes in the genomes, such as the presence of secondary metabolite-encoding genes and other proteins that are involved in the lysis of pathogenic microorganisms, determines the synthesis and secretions of volatile organic compounds [123],[124]. Endophytic bacteria that can produce volatile compounds are vital, as they enhance and improve the immunity of their host plants and would be formulated for the production of biopesticides that are environmentally healthy. For example, the tomato endophytic bacteria *B. proteolyticus*, *E. asburiae*, *E. cloacea*, *B. thuringiensis*, *B. nakamurai and B. pseudomycoides* produce bioactive compounds that facilitate the inhibition of *Botrytis cinerea*, a fungal pathogen for fresh fruits and vegetables [82]. *Bacillus amylolicefaciens* ALB629 and UFLA285 were found to secrete 3-methylbutanoic acid and 2-methylbutanoic acid, which have been suggested to have inhibited the development of anthracnose disease (*Colletotrichum lindemuthianum*) by inhibiting fungal mycelial growth and spores in *Phaseolus vulgaris* L. (common bean) [81].

### 3.5. Production of lytic enzymes

The most notable enzymes produced by the endophytic bacteria are β-1,3-glucanases, protease, cellulase, extracellular chitinase and laminarinase [125]–[127]. Production and the whole process of regulating the lytic enzymes involve the GacA/GacS or GrrA/GrrS regulatory systems and colony phase variation [104]. Enzymes lyse fungal hyphal tips and degrade any acids that might be produced by fungal pathogens [128]. Enzymes help bacteria to act as parasites for fungal pathogens and sometimes even break their spores and reduce germination [129],[130]. For example, *Bacillus pumilis* JK-SX001 is reported to secrete extracellular cellulase and protease enzymes which inhibit pathogenic fungi such as *Phomopsis macrospora*, *Cytospora chrysosperma and Fusicoccum aesculi* [76]. In another study, root endophytes *Pseudomonas poae* JA01, *Bacillus* sp. GS07 and *Paenibacillus polymyxa* GS01 were found to exhibit cellulolytic enzyme activity that aids in inhibiting the growth of fungal pathogens such as *P. ultimum*, *F. oxysporum*, *P. capsica and R. solani*, which cause notable diseases [77]. Endophytic bacteria *P. aeruginosa* and *Pseudomonas pseudoalcaligenes* were demonstrated to secrete β-1,3-glucanase and catalase in paddy and assist in the development of preformed defense against pathogenic fungi *Pyricularia grisea* that cause fungal blast [131]. The presence of endophytic bacteria in a host has also been reported to induce defense genes that encode for catalase, β-1,3-glucanase and other defense proteins in a host plant [132]. Therefore, endophytic bacteria that secrete defensive enzymes contribute to the innate immunity that is based on the preformed and induced defense responses [133].

**Figure 1.**
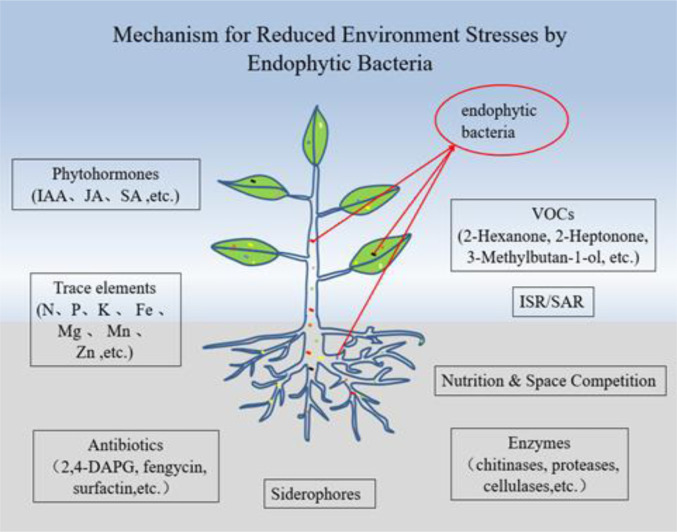
Summary of roles of endophytic bacteria in promoting plant growth and the biological control of plant pathogens.

## 4. Challenges and opportunities

Recently, due to rapid population growth, industrialization and intensive agriculture being on the rise, many plant growers have chosen to apply synthetic fertilizers and pesticides excessively to generate high yields and incur more profits at the expense of human, animal and environmental health. Given the foregoing, more interventions that are free from synthetic chemicals are needed. To achieve this, one way is through the use of beneficial microorganisms harbored by plants. To date, many plants that inhabit beneficial bacteria communities are yet to be explored, thus presenting a gap that needs to be closed for the functions and applications of endophytes to be utilized. Therefore, more research efforts are needed to explore and increase the use of endophytic microorganism communities that have the potential to be alternatively used in agriculture and environmental protection.

Endophytic bacteria are an attractive source of nutrition elements to be used as an alternative to chemical fertilizers. However, several gaps in research and utilization still exist. For example, many research studies have shown that endophytic bacteria fix atmospheric nitrogen gas into a usable form by plants. However, not so many studies have been done on the other two major nutrients, which are phosphorous and potassium. Endophytic bacteria solubilize trace elements such as iron and zinc. However, their ability to solubilize and make other crucial minor elements (e.g., manganese and molybdenum) available for plant utilization has not been made clear or fully utilized. While it is not deniable that much of the research has been concentrated on the ability of endophytes to synthesize and produce indole-3-acetic acid, further research needs to be focused on other hormones, such as zeatin, abscisic acids and gibberellic acids, as very few data are available on the ability of endophytic bacteria to produce these crucial plant growth regulators or their influence on plant growth and development. Therefore, efforts to research individual endophytic bacterial traits will aid in the development of more bioproducts than those presently available for growers. Bioformulations, as has been reported by other researchers, are “easy to deliver, able to enhance plant growth and stress resistance, increase plant biomass and yield and open the way for technological exploitation and marketing” [134].

Several of the important biocontrol traits that endophytic bacteria have are yet to be thoroughly discovered, explored or documented. In addition, several experiments show that most researchers use the dual culture method to screen the antagonistic ability of biocontrol bacterial agents. However, this method can result in the slow discovery of new biocontrol agents that can inhibit the growth of plant pathogens without showing any inhibitory effect in dual plate culture. Furthermore, the ability of endophytic bacteria to control novel plant pathogens is not known. In addition, the commercialization of biocontrol products has been very slow and limited, and it requires much attention to fully understand both the basic and advanced applications of endophytic biocontrol traits [135]. There has also been a lack of field results to demonstrate the important effectiveness of biocontrol bacteria and, as a result, there has been limited development of bioformulations of these bacteria into biopesticides. For example, the utilization of *Streptomyces* bacteria for biocontrol has been minimal as compared to the potential and ability to exhibit biocontrol properties that affect various plant pathogens [136]. Based on these challenges, thorough research needs to be conducted on the individual antibiotics, lytic enzymes and volatile compounds in terms of their synthesis and mechanism of action against plant pathogens. In addition, full utilization of the knowledge and the use of omics technological tools and other molecular biology-related studies, such as genomics, epigenetics, metabolomics and proteomics, would help to discover and understand the whole concept of biocontrol agents and their applications in agriculture and plant protection.

## 5. Conclusion

Interactions between plants and microorganisms have major influences on the environment. Of importance is the interaction between plants and their bacterial endophytes. Endophytic bacteria become part of the plant and help their hosts to overcome abiotic stresses by ensuring nutrition uptake, fixing nitrogen and solubilizing phosphates, potassium, zinc and other important trace nutrition elements. In addition, endophytes synthesize and control plant hormones such as indole-3-acetic acids, ethylene, zeatin, abscisic acids and gibberellic acids. Many bacterial endophytes can exhibit biocontrol traits that would become valuable products, including siderophores, antibiotics, volatile organic compounds and lytic enzymes. There are many opportunities to explore both already identified and unidentified endophytic bacteria to maximize their applicability in plant growth and protection. Many of the bacterial endophytes and their secretion could be commercially formulated for use on a wider scale. Finally, the existing gaps identified could be closed by furthering research on endophytes, ensuring efficient collaborations between researchers and growers and making use of our knowledge of omics and other biotechnological tools. In conclusion, endophytic bacteria represent a set of untapped agents that have the potential to replace the overuse of synthetic chemicals and enhance plant health and productivity.
